# CodY Is a Global Transcriptional Regulator Required for Virulence in Group B *Streptococcus*

**DOI:** 10.3389/fmicb.2022.881549

**Published:** 2022-04-28

**Authors:** Angelica Pellegrini, Germana Lentini, Agata Famà, Andrea Bonacorsi, Viola Camilla Scoffone, Silvia Buroni, Gabriele Trespidi, Umberto Postiglione, Davide Sassera, Federico Manai, Giampiero Pietrocola, Arnaud Firon, Carmelo Biondo, Giuseppe Teti, Concetta Beninati, Giulia Barbieri

**Affiliations:** ^1^Department of Biology and Biotechnology “Lazzaro Spallanzani,” University of Pavia, Pavia, Italy; ^2^Department of Human Pathology and Medicine, University of Messina, Messina, Italy; ^3^Department of Molecular Medicine, University of Pavia, Pavia, Italy; ^4^Institut Pasteur, Université de Paris, CNRS UMR 6047, Unité Biologie des Bactéries Pathogènes à Gram-positif, Paris, France; ^5^Charybdis Vaccines Srl, Messina, Italy

**Keywords:** group B *Streptococcus*, *Streptococcus agalactiae*, CodY, Srr2, bacterial meningitis, RNA-Seq, global regulation of gene expression, pathogenesis

## Abstract

Group B *Streptococcus* (GBS) is a Gram-positive bacterium able to switch from a harmless commensal of healthy adults to a pathogen responsible for invasive infections in neonates. The signals and regulatory mechanisms governing this transition are still largely unknown. CodY is a highly conserved global transcriptional regulator that links nutrient availability to the regulation of major metabolic and virulence pathways in low-G+C Gram-positive bacteria. In this work, we investigated the role of CodY in BM110, a GBS strain representative of a hypervirulent lineage associated with the majority of neonatal meningitis. Deletion of *codY* resulted in a reduced ability of the mutant strain to cause infections in neonatal and adult animal models. The observed decreased *in vivo* lethality was associated with an impaired ability of the mutant to persist in the blood, spread to distant organs, and cross the blood-brain barrier. Notably, the *codY* null mutant showed reduced adhesion to monolayers of human epithelial cells *in vitro* and an increased ability to form biofilms, a phenotype associated with strains able to asymptomatically colonize the host. RNA-seq analysis showed that CodY controls about 13% of the genome of GBS, acting mainly as a repressor of genes involved in amino acid transport and metabolism and encoding surface anchored proteins, including the virulence factor Srr2. CodY activity was shown to be dependent on the availability of branched-chain amino acids, which are the universal cofactors of this regulator. These results highlight a key role for CodY in the control of GBS virulence.

## Introduction

Group B *Streptococcus* (GBS, *Streptococcus agalactiae*) is the leading cause of sepsis and meningitis in neonates (Thigpen et al., 2011; Okike et al., 2014). Maternal vaginal colonization during pregnancy represents the principal risk factor for GBS transmission to the newborn through *in utero* ascending infections or aspiration of contaminated amniotic or vaginal fluids during delivery. Vertically acquired neonatal infections lead to early-onset (0–7 days of life) invasive disease manifesting as pneumonia that rapidly progresses to sepsis (Edmond et al., 2012; Patras and Nizet, 2018). GBS can also cause late-onset disease that manifests between 7 and 90 days of life with bacteremia and a high complication rate of meningitis (Tazi et al., 2019).

Group B *Streptococcus* is capable of causing these diverse clinical manifestations thanks to its capacity to invade different host niches and adapt to various environmental conditions. This versatility is made possible by the activity of several transcriptional regulators which, in response to environmental signals, control the expression of proteins involved in nutrient acquisition, adhesion, virulence, and immune evasion (Rajagopal, 2009; Thomas and Cook, 2020).

CodY is a global transcriptional regulator highly conserved in nearly all low-G+C Gram-positive bacteria, including the genera *Bacillus*, *Lactococcus*, *Streptococcus*, *Listeria*, *Staphylococcus, Clostridium*, and *Clostridioides* (Guédon et al., 2005; Lemos et al., 2008; Dineen et al., 2010; Kreth et al., 2011; Lobel et al., 2012; Belitsky and Sonenshein, 2013; Brinsmade et al., 2014; Feng et al., 2016; Waters et al., 2016; Geng et al., 2018). In these organisms, CodY directly and indirectly controls the expression of hundreds of metabolic genes in response to nutrient availability (Sonenshein, 2005). In pathogens, CodY regulates also critical virulence determinants and, therefore, links nutrient availability and metabolism to pathogenesis in a species-specific manner. The nutritional status of the cell is monitored by CodY by its interaction with two ligands: branched-chain amino acids (BCAAs) (Guedon et al., 2001; Shivers and Sonenshein, 2004; Brinsmade et al., 2010) and GTP (Ratnayake-Lecamwasam et al., 2001; Handke et al., 2008). However, while BCAAs are universal cofactors of CodY, GTP was not found to be involved in CodY activation in *Lactococcus* (Petranovic et al., 2004) and *Streptococcus* species (Hendriksen et al., 2008). Once activated by binding to its cofactors, CodY binds DNA at sites characterized by a 15-nt canonical consensus binding motif “AATTTTCWGAAAATT” (den Hengst et al., 2005; Belitsky and Sonenshein, 2013; Biswas et al., 2020). As the intracellular pools of BCAAs change, the hundreds of genes whose expression is controlled by CodY are expressed in a hierarchical manner, reflecting the choice of turning on specific metabolic pathways ahead of others (Brinsmade et al., 2014; Waters et al., 2016). In many cases, the hierarchy of gene expression stems in part from the interplay between CodY and other transcriptional regulators. That is, many direct targets of CodY regulation are also controlled by other factors and the expression of several transcriptional regulators is CodY-regulated. Consequently, regulatory circuits interlinking different pathways are created (Belitsky et al., 2015; Barbieri et al., 2016).

In this work, we aimed at providing the first global analysis of the CodY regulon in GBS and at investigating the role of this regulator in controlling and coordinating metabolism and virulence in this bacterium. To this purpose, a capsular serotype III strain belonging to the hypervirulent clonal complex 17 (CC17, as defined by Multi Locus Sequence Typing analysis) was employed. This lineage is responsible for the vast majority of cases of neonatal GBS –elicited meningitis worldwide (Manning et al., 2009; Joubrel et al., 2015; Seale et al., 2016).

## Materials and Methods

### Bacterial Strains, Plasmids, and Growth Conditions

Bacterial strains and plasmids used in this work are listed in Table 1 and Supplementary Table 1, respectively. Employed primers are listed in Supplementary Table 2. GBS was cultured in Todd Hewitt (TH, Difco Laboratories) supplemented with 5 g/liter of yeast extract (THY) or in chemically defined medium (CDM, Supplementary Table 3) (Willett and Morse, 1966) at 37°C, 5% CO_2_, steady state. *E. coli* strains were cultured in Luria Bertani (LB) broth at 37°C. Antibiotics were used at the appropriate concentrations. For *E. coli*: kanamycin 50 μg/ml; erythromycin 150 μg/ml. For GBS: kanamycin 1 mg/ml; erythromycin 10 μg/ml.

**TABLE 1:** Bacterial strains used in this work.

*S. agalactiae*			
Strain	Relevant genotype	Plasmid	Source or Reference
BM110	Serotype III, ST-17, human hypervirulent clinical isolate		Tazi et al., 2010
Δ*codY*	BM110 carrying an in-frame *codY* deletion		This work
BM1102	BM110	pTCVΩP_tet_	This work
BM1103	BM110	pTCVΩP_tet__*codY*	This work
BM1104	Δ*codY*	pTCVΩP_tet_	This work
BM1105	Δ*codY*	pTCVΩP_tet__*codY*	This work
BM1106	BM110	pTCVlacZ_*livKp220*	This work
BM1107	Δ*codY*	pTCVlacZ_*livKp220*	This work
BM1114	BM110	pTCVlacZ_*livKp1-220*	This work
BM1115	Δ*codY*	pTCVlacZ_*livKp1-220*	This work

***E. coli***			

**Strain**	**Genotype**		**Source or reference**

XL-1 Blue	*recA1 endA1 gyrA96 thi-1 hsdR17 supE44 relA1 lac [F ì proAB lacI^q^ ZΔM15 Tn10 (Tet^r^)]*		Agilent
BL21 DE3	*B F– ompT gal dcm lon hsdSB(rB–mB–) λ(DE3 [lacI lacUV5-T7p07 ind1 sam7 nin5]) [malB+]K-12(λS)*		

### Strains Construction

The pG1-Δ*codY* vector used to create the Δ*codY* derivative of BM110 (Δ*codY*) was constructed by Gibson assembly (NEBuilder HiFi DNA Assembly Cloning Kit, New England Biolabs) with PCR amplified genomic regions located upstream and downstream of *codY*, using pG1_codYUpF + BM_codYFusR and BM_codYFusF + pG1_BM_codYDwR primers, and an inverse PCR fragment obtained with the pG1R and pG1F oligonucleotides on the temperature-sensitive pG1 plasmid (Mistou et al., 2009). The three PCR fragments were fused by Gibson assembly and electroporated into *E. coli* XL1 blue. The obtained pG1-Δ*codY* plasmid was verified by sequencing and then electroporated in GBS. Transformants were selected at 30°C on TH plates supplemented with erythromycin. Plasmid integration and excision were performed as previously described (Biswas et al., 1993). The resulting in-frame deletion of *codY* on genomic DNA was verified by Sanger sequencing using external primers COH1_1525FUp and COH1_1527RDw.

To complement the *codY* deletion, the *codY* gene was amplified with oligonucleotides pTCV_codYF_Bam and pTCV_codYR_Pst, using BM110 chromosomal DNA as template. The obtained fragment was cloned between the *Bam*HI and *Pst*I sites of the pTCVΩP_tet_ vector (Buscetta et al., 2016). The resulting pTCVΩP_tet__*codY* plasmid was verified by sequencing and used to electroporate wild-type (WT) and Δ*codY* strains, thus obtaining BM1103 and BM1105, respectively (Table 1). Two control strains (BM1102 and BM1104) were prepared by electroporation of the empty pTCVΩP_tet_ plasmid into WT and Δ*codY* strains. Transformants were selected on TH agar plates supplemented with kanamycin.

### Construction of *lacZ* Transcriptional Fusions and β-Galactosidase Assays

To prepare the pTCV-*lacZ*_*livK*p_220_ plasmid (Supplementary Table 1), a 269 bp fragment comprising the regulatory region and the first 27 nucleotides of the *livK* gene was amplified with primers livKp220F and livKp220R using the BM110 chromosomal DNA as template. The obtained amplicon was inserted by Gibson assembly between the *Eco*RI and *Bam*HI restriction sites of plasmid pTCV-*lacZ* (Poyart and Trieu-Cuot, 2006), upstream of the *lacZ* gene.

To create pTCVlacZ_*livKp1-220*, a 196 bp product containing the 5′ part of the *livK* regulatory region was amplified by using oligonucleotides livKp220F and mutagenic oligonucleotide livKp1R. A 125 bp fragment comprising the 3′ part of the regulatory region and the first 27 bp of the *livK* coding sequence was synthesized by using mutagenic oligonucleotide livKp1F and livKp220R as reverse primer. The two mutagenized, partially overlapping (52 bp overlap) PCR products and the *Eco*RI/*Bam*HI digested pTCV-*lacZ* plasmid were then fused by Gibson using the NEBuilder HiFi DNA Assembly Cloning Kit (New England Biolabs).

β-galactosidase specific activity was determined as previously described (Trespidi et al., 2020).

### Time-Lapse Microscopy and Single-Cell Image Analysis

Agarose pads (Young et al., 2011) spotted with 5 μl of a 1:10 dilution of GBS cell cultures collected at mid-log phase of growth (OD600 0.5 in THY medium) were flipped and transferred to an imaging dish sealed with parafilm. Time-lapse imaging was performed using a Leica DMi8 widefield microscope, equipped with a 100× oil immersion objective (Leica HC PL Fluotar 100×/1.32 OIL PH3), a Leica DFC9000 sCMOS camera and driven by Leica LASX software. Experiments were performed using an environmental microscope incubator set at 37°C and bacteria were imaged in phase contrast, every 5-min and up to 6 h. Manual segmentation of individual cells and analysis of image stacks were performed using the ImageJ 1.52a software, as previously described (Manina et al., 2015). Data were analyzed using Prism 9.

### RNA Preparation and Quantitative Real-Time-PCR

Group B *Streptococcus* total RNA was extracted from cells collected at mid-exponential phase of growth using the Quick-RNA Fungal/Bacterial Miniprep Kit (Zymo Research) as per the manufacturer’s instructions. Traces of genomic DNA were removed from samples using the Turbo DNA-free DNase treatment and removal kit (Ambion). Reverse transcription and quantitative real-time PCR (qRT-PCR) experiments were performed in a single step using the iTaq Universal SYBR Green One-Step Kit (Bio-Rad). The reactions were performed in 20 μl volumes using 4 ng of DNAse I treated RNA and 400 nM primers targeting *livK*, *braB*, *brnQ*, and *gyrA* (used as reference gene).

### RNA-Sequencing and Analysis

RNA-Seq was performed on four independent biological replicates for each strain. rRNA was depleted using the QIAseq FastSelect –5S/16S/23S Kit (QIAGEN). RNA was sequenced using Illumina sequencing technology (BMR-Genomics, Padua). For RNA-Seq data analysis, raw reads were quality checked using FASTQC^1^ and processed by Trimmomatic (Bolger et al., 2014) to trim the adaptor sequences and remove low-quality reads. Clean reads were mapped onto the reference genome of *Streptococcus agalactiae* BM110 (Accession: NZ_LT714196.1) using Bowtie2 (Langmead, 2010). To quantify the known transcripts, the alignment results were input into featureCounts (Liao et al., 2014). Lastly, the R package DESeq2 (Love et al., 2014) was used to test for differential expression. We defined genes as differentially expressed using the following criteria: | Log2 Fold Change| ≥ 1 and adjusted *p*-value FDR < 0.05. Prediction of orthologous groups was performed using COGnitor (Tatusov, 2000).

### Mammalian Cell Culture and Epithelial Cell Adhesion Assays

A549 cells and HeLa cells were routinely grown in 75 cm^2^ flasks in Dulbecco’s modified Eagle’s medium (DMEM) supplemented with 10% fetal bovine serum (FBS) at 37°C in 5% CO_2_. Cells were seeded at 2 × 10^5^ cell density per well in 24-well tissue culture plates and cultured in DMEM without antibiotics for 24 h. Bacteria grown to the mid-log phase were added to confluent monolayers at a multiplicity of infection of 10. After a 2-h incubation, monolayers were washed three times with PBS to remove the non-adherent bacteria, lysed, and serial dilutions of the cell lysates were plated to enumerate cell-associated bacteria. Percent of adhesion of each strain was calculated as follows (number of CFUs on plate)/(number of CFUs of initial inoculum) × 100. Percentage of adhesion was normalized to the WT strain, set at 100%.

### Biofilm Formation Assay

For bacterial biofilm formation assays, a 1:20 dilution of an overnight culture grown in TH broth supplemented with 1% glucose and the appropriate antibiotic was used to inoculate (100 μl/well) a 96-well Tissue Culture Treated plate (16 technical replicates per strain). Non-adherent bacteria were removed by washing with PBS after 6 h of incubation at 37°C, 5% CO_2_. Crystal violet staining was performed as previously described (Trespidi et al., 2021) after 19 h of incubation in TH + 1% glucose medium (37°C, 5% CO_2_). Biofilm growth was evaluated by reading absorbance at 595 nm and normalizing the obtained value to the OD600 of the culture in the well.

For confocal microscopy analysis of biofilms, bacterial overnight cultures in TH broth supplemented with 1% glucose and the appropriate antibiotic were diluted in the same medium to an OD_600_ 0.05 (about 1 × 10^7^ CFU/mL) before being added to a four-well Nunc Lab-Tek II Chambered Coverglass. Non-adherent cells were removed after 6 h. After overnight growth, biofilms were washed twice with PBS and stained with 5 μM Syto 9 (Invitrogen). Cells were imaged with a Leica TCS SP8 confocal microscope equipped with a Leica DMi8 inverted microscope, a tunable excitation laser source (White Light Laser, Leica Microsystems, Germany), and driven by Leica Application Suite X, ver. 3.5.6.21594, using a 63× oil immersion objective (Leica HC PL APO CS2 63X/1.40). Images were acquired using a 488 nm laser line as an excitation source, and the fluorescence emitted was collected in a 500–540 nm range for Syto 9 as previously described (Trespidi et al., 2020). Biofilm images were visualized and processed using ImageJ. Biofilm parameters were measured using the COMSTAT 2 software (Heydorn et al., 2000). All confocal scanning laser microscopy experiments were performed three times and standard deviations were measured.

### Cloning, Overproduction, and Purification of CodY

The *codY* CDS was amplified with primers pET_GBS_codYF and pET_GBS_codYR. The obtained fragment was inserted by Gibson assembly between the *Eco*RI and *Bam*HI sites of plasmid pET28a. The CodY protein with an N-terminal 6X-His tag was produced in *E. coli* BL21 DE3 cells by IPTG (0.5 mM) induction at 28°C overnight. CodY protein was purified as previously described (Alfeo et al., 2021), and protein concentration was measured by Bradford protein assay (Bio-Rad).

### Fragments Labeling and Electrophoretic Mobility Shift Assay

To be used as probes in gel-shift experiments, PCR products containing the regulatory region of the *livK* gene were amplified using the appropriate pTCV-*lacZ* derivative plasmid as template and the 5’FAM labeled, vector-specific primers Vlac1-FAM and Vlac2-FAM.

FAM-labeled fragments (50 nM) were incubated with increasing concentrations of CodY and electrophoretic mobility shift assays were performed as previously described (Barbieri et al., 2015). When indicated, BCAAs were added to the final concentration of 10 mM in the CodY-binding reaction mixture. Ten mM each isoleucine, leucine, and valine were also added to the 5% non-denaturing Tris-Glycine polyacrylamide gel and electrophoresis buffer.

### Mouse Infection Models

In the neonatal model, 48-h-old mice of both sexes were inoculated subcutaneously with 8 × 10^4^ CFU of the WT or the Δ*codY* strain, as previously described. Mice showing signs of irreversible disease, such as diffuse redness spreading from the infection site, were humanely euthanized. In the adult model of GBS sepsis, 8 week-old female mice were inoculated intraperitoneally with 5 × 10^8^ CFU of the WT or the Δ*codY* strain, as previously described (Biondo et al., 2014). In the meningitis model, 8 week-old female mice were inoculated intravenously 1 × 10^9^ CFU of WT or the Δ*codY* strain, as previously described (Lentini et al., 2018). Mice showing signs of irreversible disease, such as prolonged hunching, inactivity, or neurological symptoms were humanely euthanized. In further experiments, mice were euthanized at 16 h after challenge and bacterial burden was determined in organ homogenates, as previously described (Famà et al., 2020).

### *In vivo* and *in vitro* Cytokine Induction

Female mice of 8 weeks of age were infected intraperitoneally with 1 × 10^9^ CFU of the WT or the Δ*codY* strain. Mice were treated at 30 min post-challenge with penicillin (500 IU i.p.) to prevent bacterial overgrowth. Peritoneal lavage fluids were collected at the indicated times and analyzed for cytokine levels as previously described (Mohammadi et al., 2016). For *in vitro* cytokine induction, bone marrow-derived macrophages were obtained from 8-week-old female mice and cultured in the presence of M-CSF as previously described (Lentini et al., 2021). Macrophage cultures were then stimulated for 1 h with GBS grown to the late exponential phase at the indicated multiplicities of infection (MOI). Cultures were then treated with penicillin and gentamycin (100 IU and 50 μg/ml) to kill extracellular bacteria and supernatants were collected at 18 h after culture, as previously described (Lentini et al., 2021). Cytokine levels were measured in peritoneal lavage fluid samples or culture supernatants by ELISA, using Mouse TNF-alpha DuoSet ELISA DY410, Mouse IL-1 beta/IL-1F2 DuoSet ELISA DY401, Mouse CXCL2/MIP-2 DuoSet ELISA DY452, Mouse CXCL1/KC DuoSet ELISA DY453 (R&D Systems).

## Results

### CodY Is Required for *in vivo* Virulence of Group B *Streptococcus*

**FIGURE 1:**
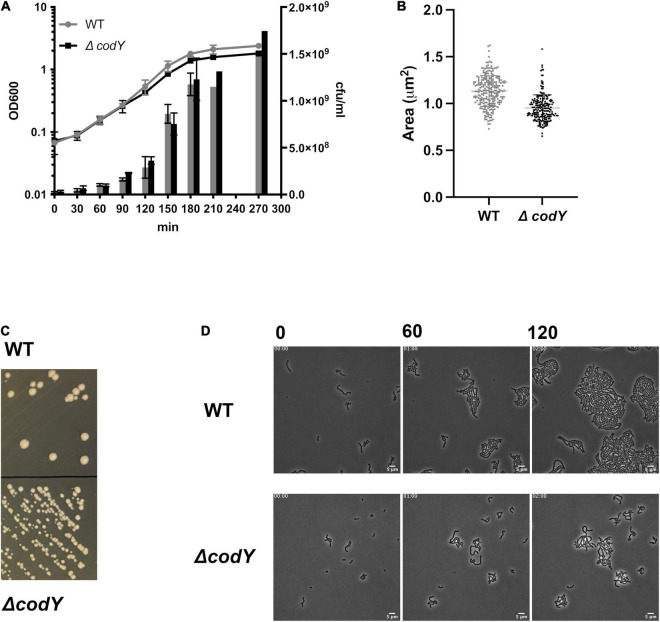
Deletion of *codY* does not affect cell growth but is associated to a smaller cell size and colony morphology. **(A)** Growth of the BM110 strain (WT) and of the isogenic *codY* deletion mutant (Δ*codY*) in THY rich medium, evaluated as absorbance at OD_600_ (left axis) and cfu/ml (right axis). Data are the average ± SD of two independent experiments **(B)**. Single cell area measurements (μm^2^) of 50 cells of each strain. Reported data refer to two independent experiments. Statistically significant differences are indicated (Welch’s *t*-test). **(C)** Colonies of the WT and Δ*codY* strains grown on THY agar plates. **(D)** Contrast-phase image stacks of BM110 and Δ*codY* cells during time-lapse microscopy. Scale bar is 5 μm.

A marker-free, in-frame deletion of the *codY* gene was created by allelic replacement in the CC17 wild-type strain BM110 (WT). The resulting Δ*codY* mutant showed no growth defects in rich THY liquid medium (Figure 1A). However, Δ*codY* cells showed a 10% reduced cell size and formed smaller colonies compared to the WT strain (Figures 1B–D), similarly to what was previously observed in *codY*-deleted mutants in other bacteria (Majerczyk et al., 2008; Geng et al., 2018).

**FIGURE 2:**
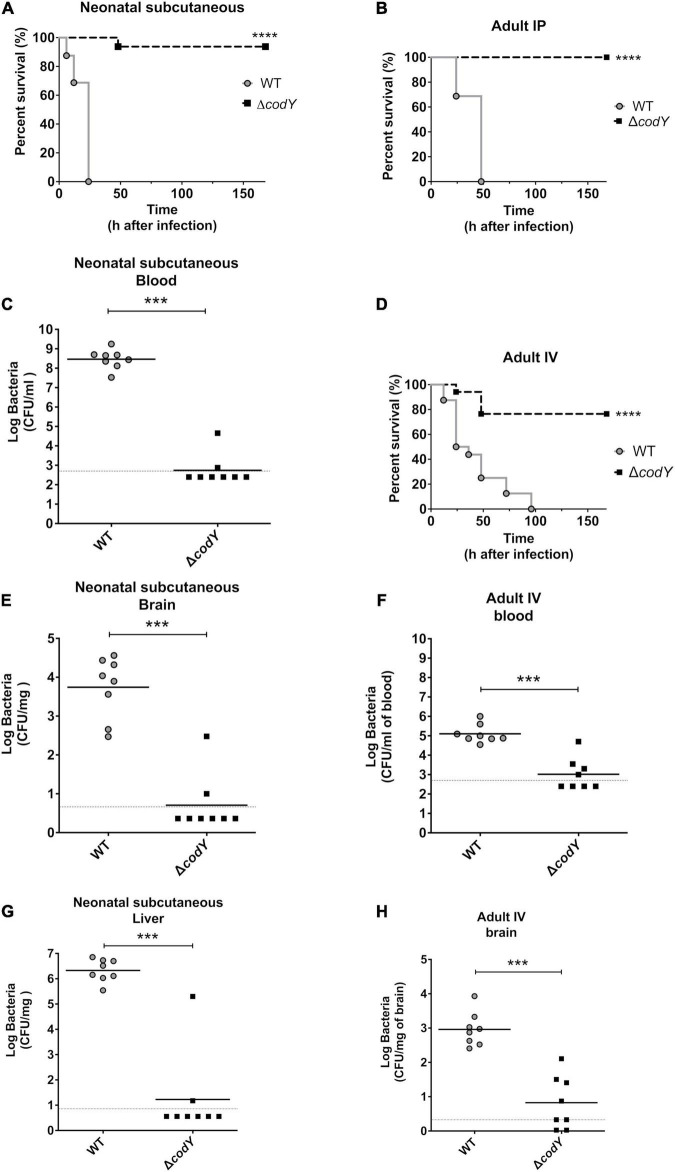
*CodY* is necessary during neonatal and adult infections. **(A)** Lethality of newborn mice (8 per group) infected subcutaneously with 8 × 10^4^ of the WT strain or the Δ*codY* mutant. **(C,E,G)** Bacterial burden in the indicated organs of newborn mice infected in the same experimental condition as in panel **(A)**; horizontal bars indicate mean log CFU values. **(B)** Lethality of adult mice (8 per group) infected intraperitoneally (IP) with 5 × 10^8^ of the WT strain or Δ*codY* mutant. **(D)** Lethality of adult mice (8 per group) infected intravenously (IV) with 1 × 10^9^ CFU. **(F,H)** Bacterial burden in the indicated organs of adult mice infected in the same experimental condition as in panel **(D)**; horizontal bars indicate mean log CFU values. ***p < 0.001; ****p < 0.0001 by the Kaplan Meier (panels **A,B,D**) or Mann Whitney test (panels **C, E–H**).

To assess the *in vivo* impact of CodY on the ability of GBS to sustain infection, we determined the virulence properties of the Δ*codY* strain in several models of infection that closely mimic features of human infections (Magliani et al., 1998; Cusumano et al., 2004). In the first murine model of neonatal GBS sepsis, bacteria replicate at the inoculation site and spread systemically to the blood and distant organs. Newborn mice infected subcutaneously with the WT strain showed signs of irreversible infection within the first 24 h after challenge and were humanely euthanized. In contrast, nearly all neonates infected with the Δ*codY* strain survived and remained in good conditions until the end of the experiment (Figure 2A). In further studies, newborn mice were infected as above, and the organs were collected at 14 h after challenge. As shown in Figures 2C,E,G, considerable bacterial burden was detected in the blood, brain, and liver of all animals infected with WT GBS, while low bacterial numbers or no bacteria were present in the organs from mice infected with the *codY*-deleted strain. These data indicated that, in the absence of CodY, GBS is unable to replicate locally *in vivo* and to spread hematogenously to distant organs. Since GBS infections are being increasingly reported in adults, we sought to confirm the data obtained in newborn mice in an adult sepsis model. As shown in Figure 2B and Supplementary Figures 1A–E, all adult mice intraperitoneally inoculated with the *codY*-deleted strain survived while all mice infected with WT bacteria succumbed to overwhelming infection, confirming the results obtained in neonates. In view of the clinical importance of meningoencephalitis in the context of CC17 GBS infection, we also looked at the role of CodY in the ability of GBS to cross the blood-brain barrier using a meningoencephalitis model in which bacteria are inoculated intravenously. Under these conditions, the Δ*codY* mutant displayed a considerably decreased ability to persist in the blood and to cause lethal encephalitis compared to WT bacteria (Figures 2D,F,H).

### Deletion of CodY Does Not Impact the Host Cytokine Response to Group B *Streptococcus* Infection

To investigate whether the reduced virulence of GBS in the absence of CodY could be related to altered induction of pro-inflammatory cytokines, we used a sepsis model in which mice are infected intraperitoneally and cytokine levels are measured in peritoneal lavage fluid samples at different times after challenge. To avoid bacterial overgrowth, penicillin was administered at 30 min post-challenge. Under these conditions, TNF-α, IL-1β, Cxcl1, and Cxcl2 levels rapidly increased, to reach peak levels at 3 h after challenge with a WT strain (Supplementary Figures 2A–D). However, similar cytokine levels were detected in mice infected with the WT and Δ*codY* strains. Similarly, no differences were detected in TNF-α or IL-1β induction in peritoneal macrophages stimulated with the two strains (Supplementary Figures 2E,F).

### CodY Contributes to Group B *Streptococcus* Adhesion to Epithelial Cells

**FIGURE 3:**
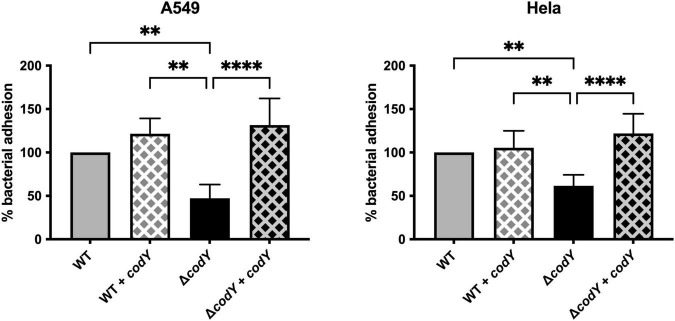
CodY controls GBS adhesion to epithelial cells. Cell adhesion was quantified using HeLa and A549 immortalized cell lines. The WT and the Δ*codY* mutant contain the empty vector pTCVΩP_tet_ or the complementing vector pTCVΩP_tet__*codY* (+ *codY*). The percent of adhesion of each strain was calculated relative to the initial inoculum and was normalized to the adhesion of the WT strain, set as 100%. Represented data are the average ± SD of six independent experiments, each performed in duplicate. Statistically significant differences are indicated (One-way ANOVA). ^**^p < 0.01, ^****^p < 0.0001.

Adhesion to host cells and tissue colonization are necessary for the establishment of a successful infection. Deletion of *codY* resulted in a 50% decrease in adherence to human epithelial cervix adenocarcinoma (HeLa) and human epithelial lung carcinoma (A549) cell lines compared to the WT strain (Figure 3). Complementation of *codY* deletion by plasmid-mediated expression of *codY* under the control of the constitutive Ptet promoter restored adhesion to levels similar to the WT strain. Plasmid-mediated CodY expression in the WT strain did not affect the adhesion ability of the parental strain.

### CodY Controls the Ability of BM110 to Form Biofilms

**FIGURE 4:**
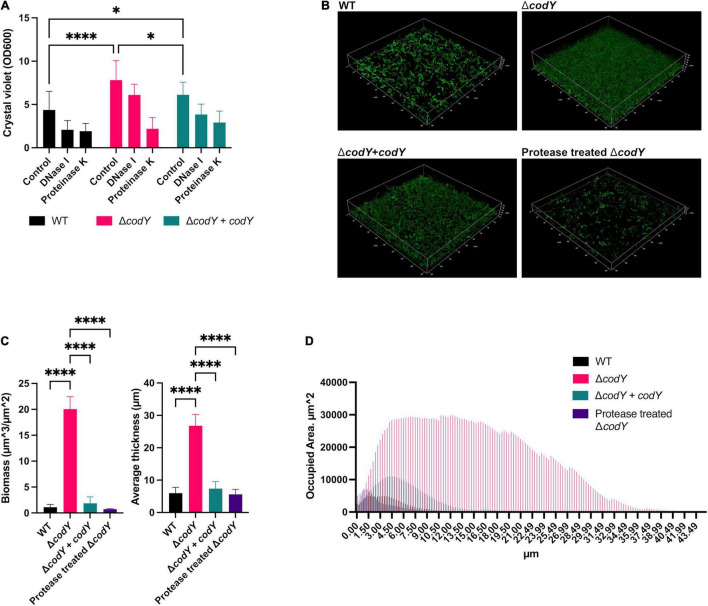
CodY inhibits biofilm formation. **(A)** Biofilm biomass was measured by crystal violet staining after static culture for 19 h. The WT and the Δ*codY* strain contain the empty vector pTCVΩP_tet_ or the complementing vector pTCVΩP_tet__*codY* (+ *codY*). The role of eDNA and extracellular proteins as biofilm structural components was assessed by individual treatments with DNase I and proteinase K, respectively. Represented data are the average ± SD of two independent experiments, each performed in 8 replicates. Statistically significant differences are reported (two-way ANOVA). **(B)** Confocal laser scanning microscopic images of biofilms formed by WT, Δ*codY* and Δ*codY + codY* GBS strains grown for 19 h in Nunc*™* Lab-Tek*™* II Chambered Coverglass. The effect of treatment with proteinase K on the Δ*codY* biofilm is reported. **(C,D)** Analysis of biofilm properties by COMSTAT 2. Measures of total biomass, average thickness **(C)** and % of the area occupied by biofilm distribution **(D)**. Data are the average ± SD of the results from three independent replicates. : *p < 0.05, ^****^p < 0.0001 (one-way ANOVA test).

The role of CodY in the ability of GBS to form biofilms was evaluated by crystal violet staining (Figure 4A) and confocal laser scanning microscopy (Figure 4B). While the WT strain formed a weak biofilm, the *codY*-deleted mutant formed a thicker, more compact biofilm able to completely cover the surface of the well and of the chambered coverglass (Figures 4B–D). The biofilm-forming ability was significantly reduced after complementation of the *codY* deletion. Eradication experiments revealed that biofilms formed by the Δ*codY* mutant were strongly reduced by treatment with proteinase K, while DNAse I was less effective against the biofilm biomass (Figure 4A). These results suggest that extracellular proteins are a major constituent of the Δ*codY* biofilm.

### CodY Is a Global Regulator of Gene Expression in Group B *Streptococcus*

**FIGURE 5:**
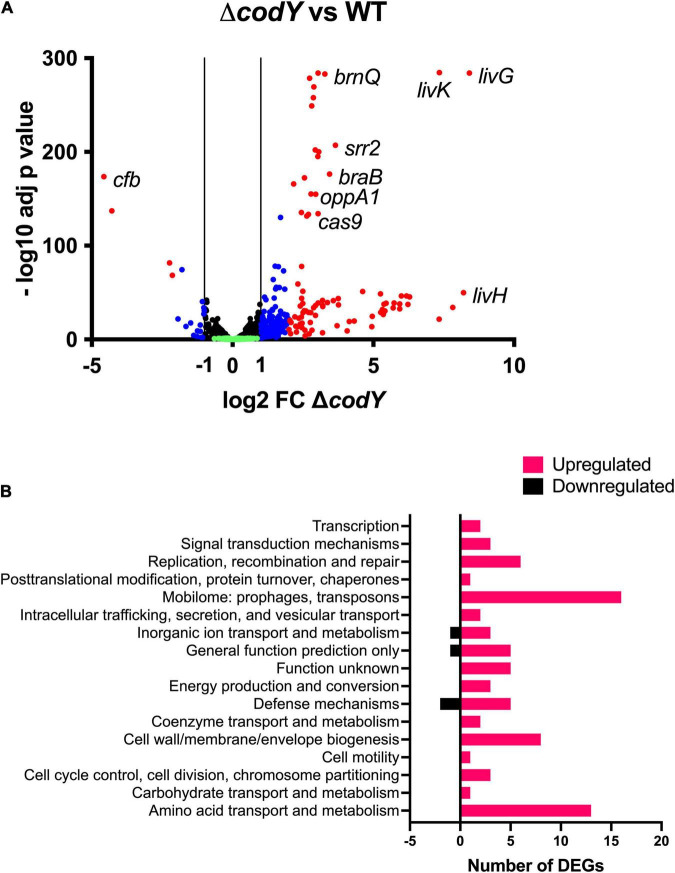
**(A)** Volcano plot of the Δ*codY* transcriptome at mid-exponential phase in THY. Each dot represents a gene with its RNA-seq fold change and adjusted *p*-value calculated from four independent replicates. Genes showing a significant differential expression in the Δ*codY* strain (adjusted *p*-value < 0.05) are represented in blue (–2 ≤ log2FC ≤ –1 or 1 ≤ log2FC ≤ 2) and red (log2FC < –2 or log2FC > 2) according to their fold change relative to the WT strain. Black dots represent genes whose expression is not affected by *codY* deletion (–1 < log2FC < 1; adjusted *p*-value < 0.05). Green dots correspond to non-significant (adjusted *p*-value > 0.05) differentially transcribed genes. **(B)** COGs associated to downregulated and upregulated genes.

To determine the transcriptional changes associated with *codY* deletion, an RNA-Seq experiment was performed on WT and Δ*codY* bacteria during exponential growth in rich THY medium, i.e., under conditions of maximal CodY activity. A total of 277 genes (out of 2,128 analyzed genes) were differentially expressed at least twofold (adjusted *p*-value < 0.05) in the Δ*codY* strain, demonstrating a global regulatory role for CodY (Supplementary Datasets 1A–C). Among these, 256 genes were up-regulated (Supplementary Dataset 1B) and 21 genes were down-regulated (Supplementary Dataset 1C) in the mutant, supporting a role for CodY mainly as a repressor of gene expression (Figure 5A). Overall, fold changes associated with negative regulation were higher than those associated with positive regulation. Notably, 55% (140/256) of the over-expressed genes were located in four prophages. The 98 genes whose expression was affected by *codY* deletion at least fourfold (94 up-regulated and 4 down-regulated genes) (Supplementary Datasets 1D,E) could be classified into seventeen categories by the Cluster of Orthologous Genes (COGs) analysis (Supplementary Dataset 2 and Figure 5B). Among these, the most represented groups included genes involved in “amino acid transport and metabolism,” “cell wall/membrane/envelope biogenesis,” and “mobilome: prophages, transposons.”

Specifically, CodY-repressed genes (Supplementary Dataset 1D and Figure 5A) included those encoding BCAAs transporters (*braB*, *brnQ*, all the genes belonging to the *livK-G* operon), the (oligo)peptide permease OppA1-F, adhesins, and serine peptidases anchored to the cell wall through the LPxTG motif, as well as proteins involved in DNA replication, recombination, and repair. Interestingly, the genes of the *cas* operon, involved in adaptive immunity, were among the ones more intensely up-regulated in the Δ*codY* mutant. Notably, the operon encoding for the CC17-specific virulence factor Srr2 was over-expressed in the absence of CodY. On the contrary, the gene encoding the CAMP factor pore-forming toxin Cfb was under positive regulation by CodY (Supplementary Dataset 1E and Figure 5A).

Using the FIMO Motif Search Tool (Grant et al., 2011), the genome of BM110 was scanned to search for sequences matching the conserved AATTTTCWGAAATT CodY binding motif. One hundred and one matches were retrieved from the genomic regions located upstream of the coding sequences of the genes, using a *p*-value lower than 0.0001. At least one of these sites was located in the proximity of the coding sequence of eighteen genes differentially expressed in the Δ*codY* strain (Supplementary Datasets 1A–D and Supplementary Table 4), predicting that these genes might be targets of direct CodY-mediated regulation.

### Group B *Streptococcus* CodY Controls Gene Expression in Response to Branched-Chain Amino Acid Availability

**FIGURE 6:**
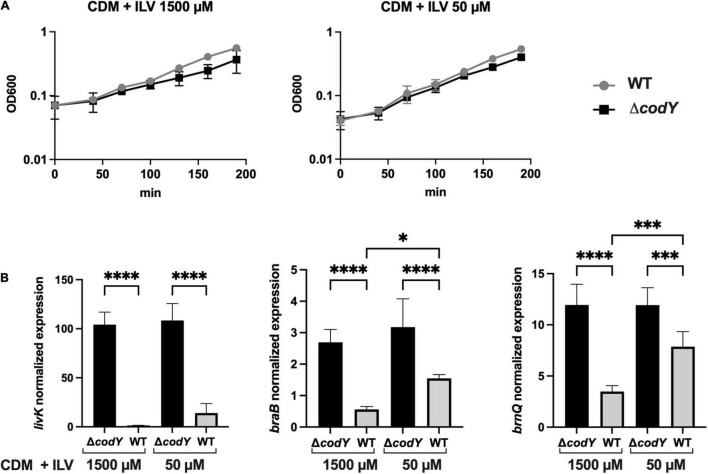
CodY activity is dependent on branched-chain amino acids availability. **(A)** Growth of the WT (gray) and Δ*codY* mutant (black) in CDM supplemented with high (1500 μM) and low (50 μM) ILV concentrations. Reported data are the average ± SD of two independent experiments. **(B)** Expression analysis by qRT-PCR of the genes *livK*, *braB*, and *brnQ* in the WT and Δ*codY* mutant grown under high and low ILV concentrations. Gene expression is normalized to the expression of the housekeeping *gyrA* gene. Asterisks denote statistically significant differences as assessed by One-way ANOVA analysis *p < 0.05, ***p < 0.001, ****p < 0.0001.

As BCAAs (isoleucine, leucine, and valine, ILV) are universal positive cofactors of CodY (Richardson et al., 2015), the expression of CodY-dependent genes is expected to change in response to the availability of these amino acids. To test this hypothesis, the expression of CodY-regulated genes was analyzed by qRT-PCR in WT and Δ*codY* cells grown to mid-log phase in CDM (Willett and Morse, 1966) containing a mix of all amino acids and supplemented with high (1,500 μM) or low (50 μM) concentrations of ILV. As GBS is unable to synthesize the precursors of most amino acids, including the BCAA (Glaser et al., 2002), ILV cannot be omitted from the growth medium. Under both conditions tested, the two strains showed similar growth kinetics, displaying approximately a two-fold increase in their doubling time compared to growth in rich, THY medium (Figure 6A). Three genes encoding BCAAs transporters and identified by RNA-Seq analysis (Supplementary Dataset 1D and Figure 5A) and qRT-PCR (Supplementary Figure 3) as subjected to different levels of CodY-mediated repression during growth in rich THY medium were included in the analysis. In the WT strain, transcription of all three genes increased when BCAA were less abundant in the defined medium, in accord with expected decrease in CodY activity. All three target genes were further and significantly over-expressed in the Δ*codY* mutant compared to the WT strain under both conditions tested, however, the extent of this overexpression was higher in *livK* (82-fold and 11-fold increase in CDM + 1500 μM and in CDM + 50 μM ILV, respectively) and lower in *braB* (3.4-fold and 2.3-fold increase in CDM + 1500 μM and in CDM + 50 μM ILV, respectively) and *brnQ* (4.8-fold and 2.8-fold increase in CDM + 1500 μM and in CDM + 50 μM ILV, respectively) (Figure 6B and Supplementary Table 5). The high level of gene expression in the Δ*codY* mutant was not affected by ILV levels. The obtained results in the WT strain suggest that, as the levels of BCAAs decrease, the expression of CodY-repressed genes increases in a gene-specific manner (Figure 6B).

### Direct Transcriptional Repression of the *livK-G* Operon by CodY

**FIGURE 7:**
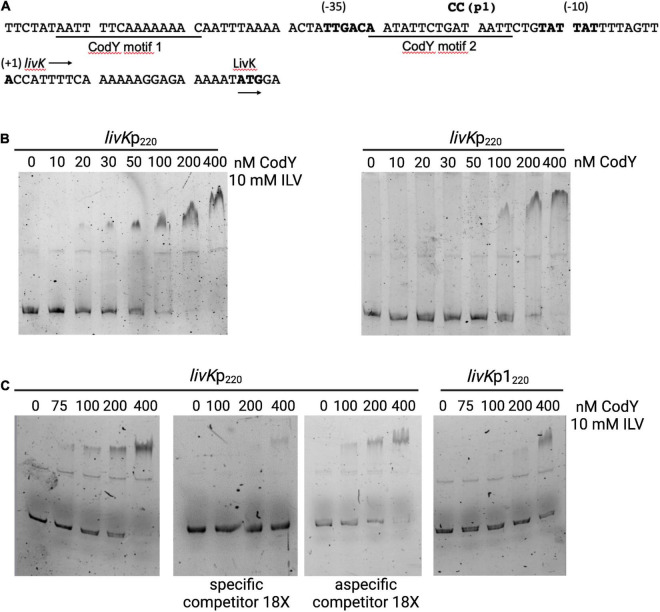
CodY binds to the *livK* promoter. **(A)** Sequence (5’ to 3’) of the *livK* regulatory region. Coordinates are reported with respect to the transcription start point (Mazzuoli et al., 2021). The transcription start site, −10 and −35 promoter regions, and the starting codon of the *livK* CDS are in boldface. The directions of transcription and translation are indicated by the horizontal arrows. The sequences of the two CodY-binding motifs are underlined. The mutated nucleotides (p1) are shown above the sequence. **(B)** Electrophoretic mobility shift assays (EMSA) for binding of CodY to the *livK* regulatory region. CodY and the WT *livK*p_220_ fragment were incubated in the presence or absence of 10 mM each isoleucine, leucine, and valine (ILV). When ILV were included in the reaction mixture, the non-denaturing polyacrylamide gel and the electrophoresis buffer were supplemented with 10 mM ILV. **(C)** EMSA performed with the WT *livK*p_220_ and mutated *livK*p1_220_ fragments. Specificity of binding was evaluated by performing the experiment with the WT fragment in the absence or presence of a specific (unlabeled *livK*p_220_ fragment) or non-specific competitor. DNA-Protein mixtures were separated on 5% non-denaturing gel in presence of 10 mM ILV.

The mechanism of CodY-mediated regulation of the *livK-G* operon, encoding an ABC-type BCAAs transporter, was investigated. Two putative CodY-binding motifs, with three and two mismatches to the consensus sequence, were identified by FIMO analysis at positions from −64 to −50 and from −31 to −17 respectively, with respect to the transcription start site (Mazzuoli et al., 2021) of the *livK* gene, the first gene of the operon (Figure 7A).

An electrophoretic-mobility shift assay (EMSA) was performed using purified CodY and a 6-carboxyfluorescein (FAM) labeled fragment encompassing the *livK* regulatory region from position −168 to +52 with respect to the transcription start site of the gene (Figure 7A). In the presence of 10 mM ILV, CodY bound the *livK* fragment with an apparent equilibrium dissociation constant (K_D_) of ≈50 nM (here, K_D_ reflects the concentration of CodY required to shift 50% of DNA fragments under conditions of vast protein excess over DNA) (Figure 7B). When ILV were omitted from the binding mixture, affinity of CodY for the *livK* regulatory region decreased (KD ≈150 nM), suggesting that BCAAs enhance CodY activity. Specificity of CodY binding was assessed by competitive and non-competitive binding assays in the presence of ILV and 18-fold excess of unlabeled specific or non-specific competitor DNA, respectively (Figure 7C).

A *lacZ* transcriptional fusion (*livK*p_220_-*lacZ*) including the region spanning from position −168 to +52 with respect to the *livK* transcription start site was constructed using the pTCV-*lacZ* plasmid (Poyart and Trieu-Cuot, 2006). Under conditions of maximal CodY activity, during the exponential phase of growth in THY medium, expression of the *livK*p_220_-*lacZ* fusion was about 400-fold higher in the *codY*-null mutant strain, BM1107, than in the WT strain, BM1106 (Table 2). A two-nucleotide substitution mutation was introduced at positions −22 and −23 with respect to the *livK* transcription start site, within the putative CodY-binding site located immediately upstream of the *livK* coding region (*livK*p1_220_-*lacZ*). The p1 mutation, aimed at decreasing the similarity of the motif to the CodY-binding consensus sequence, strongly reduced the affinity of CodY for the *livK* regulatory region (Figure 7C) and abolished CodY’s ability to repress the *livK* promoter (Table 2).

**TABLE 2:** Expression of *livK-lacZ* fusions^a^.

Strain	Relevant genotype*^b^*	Fusion genotype	β-galactosidase activity*^c^*		
			Miller Units*^a^*	%^b^	Repression^c^ ratio
BM1106	wild-type	*livK*p_220_-*lacZ*	0.47 ± 0.07	0.3	393.62
BM1107	Δ*codY*		185 ± 19.02	100.0	
BM1114	wild-type	*livKp1*_220_-*lacZ*	157.6 ± 0.35	97.3	1.03
BM1115	Δ*codY*		161.9 ± 6.86	100.0	

*^a^β-galactosidase activity is reported in Miller Units. Data are the average ± SD of two independent experiments, each performed in duplicate.*

*^b^β-galactosidase activity of each fusion in the codY-deleted strain was normalized to 100%.*

*^c^The repression ratio is the ratio of expression values for the corresponding fusions in the codY null mutant in and wild-type strain.*

## Discussion

In this study, we showed that the global transcriptional regulator CodY is essential for GBS virulence in several animal models of infection.

In low-G+C Gram-positive pathogens, this conserved transcriptional regulator coordinates metabolism and virulence in response to nutrient availability (Brinsmade, 2017). While CodY controls global metabolism in a generally conserved manner, genes involved in virulence are subjected to species-specific modes of regulation, depending on the occupied niche during infection and on the type of interaction that the bacterium establishes with the host. In *S. aureus* and *C. difficile* CodY strongly represses virulence genes, so that their expression is activated only when BCAA levels are low (Dineen et al., 2007, 2010; Majerczyk et al., 2008, 2010; Waters et al., 2016). On the contrary, in *Bacillus anthracis* and *Listeria monocytogenes* virulence is positively controlled by CodY (van Schaik et al., 2009; Château et al., 2011; Lobel et al., 2012, 2015). While understanding the function of CodY in *S. pneumoniae* is complicated by the *codY* essentiality in this important human pathogen (Caymaris et al., 2010), a role for this regulator in the control of virulence was demonstrated in other Streptococcal species (Malke et al., 2006; Lemos et al., 2008; Kreth et al., 2011; Feng et al., 2016; Geng et al., 2018).

Here, we confirmed that *codY* is not essential for the growth of GBS in complex or chemically defined liquid medium (Hooven et al., 2016) but is required *in vivo*. The reduced ability of the Δ*codY* mutant to cause infection is associated with a lower ability to disseminate, colonize host tissues, persist in blood and cause meningitis. This reduced virulence is not associated with an altered cytokine response in the host but is related to pleiotropic effects of the *codY* deletion, such as the decreased ability of the mutant strain to bind to human epithelial cells *in vitro* and the increased ability to form biofilm. Of note, while strains of the CC17, responsible for neonatal invasive infections, are generally weak biofilms formers, the ability to form strong biofilms is a common phenotype of strains able to asymptomatically colonize the host (Parker et al., 2016). As proteins appear to play a major role in promoting Δ*codY* biofilm structural stability, it is possible to speculate that surface proteins involved in bacterial adherence and encoded by genes that are repressed by CodY (e.g., Srr2, FbsB, and ScpB3) might be required for biofilm formation in GBS (Park et al., 2012).

The transcriptomic analysis in GBS strengthens the conserved role of CodY as a global regulator of metabolism, with genes encoding functions involved in the uptake of amino acids and oligopeptides subjected to the highest level of regulation. As genes required for the biosynthesis of precursors of most amino acids, including BCAAs, are missing in the genome of GBS, this bacterium relies on transporters and peptidases for amino acids metabolism (Milligan et al., 1978; Glaser et al., 2002). The capacity to take up exogenous oligopeptides is particularly important to support growth in amniotic fluid, which contains only low amounts of free amino acids (Mesavage et al., 1985; Samen et al., 2004). Notably, the majority of the genes involved in peptide and amino acid transport and metabolism that are upregulated during GBS growth in amniotic fluid (*oppA1-F* and *livK* operons, *braB*, *brnQ*, BQ8897_RS10635) are members of the CodY regulon identified in this work. As the *codY* gene itself is downregulated 11-fold during growth in amniotic fluid compared to a rich laboratory medium (Sitkiewicz et al., 2009), it might be hypothesized that the reduced levels of this repressor could be at the origin of the overexpression of peptides and amino acids transport systems in amniotic fluid.

We confirmed that the CodY response in GBS is dependent on the concentration of extracellular BCAAs which, besides being abundant amino acids in proteins, are precursors of branched-chain fatty acids, the predominant membrane fatty acids in Gram-positive bacteria (Richardson et al., 2015). CodY-mediated regulation of three genes involved in amino acid uptake (*livK*, *braB*, *brnQ*) is dependent on the level of BCAAs available in the growth medium. Therefore, as the abundance of its cofactors decreases, CodY-mediated repression of genes required for amino acid uptake is relieved. Among the analyzed genes, very low levels of *livK* expression were observed in a WT strain even under conditions of low BCAA-abundance. This result suggests that very few active molecules of CodY are sufficient to efficiently bind the regulatory region of the *livK* operon and repress its expression.

The CodY regulatory network links the metabolic status of several bacteria with the regulation of their virulence (Lobel et al., 2012; Waters et al., 2016). In GBS, CodY directly and indirectly regulates numerous genes involved in carbon and energy metabolism, cell wall and membrane biogenesis and virulence. The latter category includes surface-anchored proteins such as the Srr2 adhesin. This CC17-specific adhesin is a major virulence factor that supports the ability of GBS to cross the developing neonatal gastrointestinal epithelium and to adhere to and invade cerebral endothelial cells, thus leading to invasive infections and meningitis in neonates (Seifert et al., 2006; Six et al., 2015; Hays et al., 2019; Gori et al., 2020; Deshayes de Cambronne et al., 2021). Importantly, the transcription of the *srr2* operon and of other genes included in the CodY regulon are directly repressed by the master regulator of virulence CovR (Mazzuoli et al., 2021). In the related pathogen *S. pyogenes*, CodY represses *covR* expression allowing to counterbalance CovRS activity according to the nutritional status of the cell (Kreth et al., 2011). Noteworthy, in GBS, CovR and CodY do not control each other’s transcription (Mazzuoli et al., 2021), suggesting the existence of a different wiring between these two major regulatory pathways. A detailed investigation of the interplay between CodY and CovR regulations is necessary to define the mechanism(s) allowing a concerted regulation of virulence and metabolism in GBS.

Understanding how CodY activity is coordinated with the network of regulators controlling GBS adaptation and virulence will allow deciphering the signals and conditions governing host-pathogen interaction during colonization and infection.

## Data Availability Statement

The data presented in the study are deposited in the NCBI BioProject database (https://www.ncbi.nlm.nih.gov/bioproject/), BioProject accession number PRJNA808867.

## Ethics Statement

The animal study was reviewed and approved by the Animal Welfare Committee of the University of Messina and the Ministero della Salute of Italy (Permit number 786/2018-PR prot. 5E567.10).

## Author Contributions

GB, GP, CBe, GTe, AFi, and CBi conceived the work and designed the experiments. AP, AB, VS, GTr, FM, and SB conducted the experiments. UP and DS performed the bioinformatic analyses. GL and AFa performed *in vivo* experiments. GB and AP wrote the manuscript. All authors contributed to the article and approved the submitted version.

## Conflict of Interest

The authors declare that the research was conducted in the absence of any commercial or financial relationships that could be construed as a potential conflict of interest.

## Publisher’s Note

All claims expressed in this article are solely those of the authors and do not necessarily represent those of their affiliated organizations, or those of the publisher, the editors and the reviewers. Any product that may be evaluated in this article, or claim that may be made by its manufacturer, is not guaranteed or endorsed by the publisher.

## Footnotes

1
https://qubeshub.org/resources/fastqc
