# Supplementary figures and images for: CodY Is a Global Transcriptional Regulator Required for Virulence in Group B Streptococcus
Source: Front Microbiol. 2022 Apr 28;13:881549. doi: 10.3389/fmicb.2022.881549 (PMC9096947; doi:10.3389/fmicb.2022.881549)

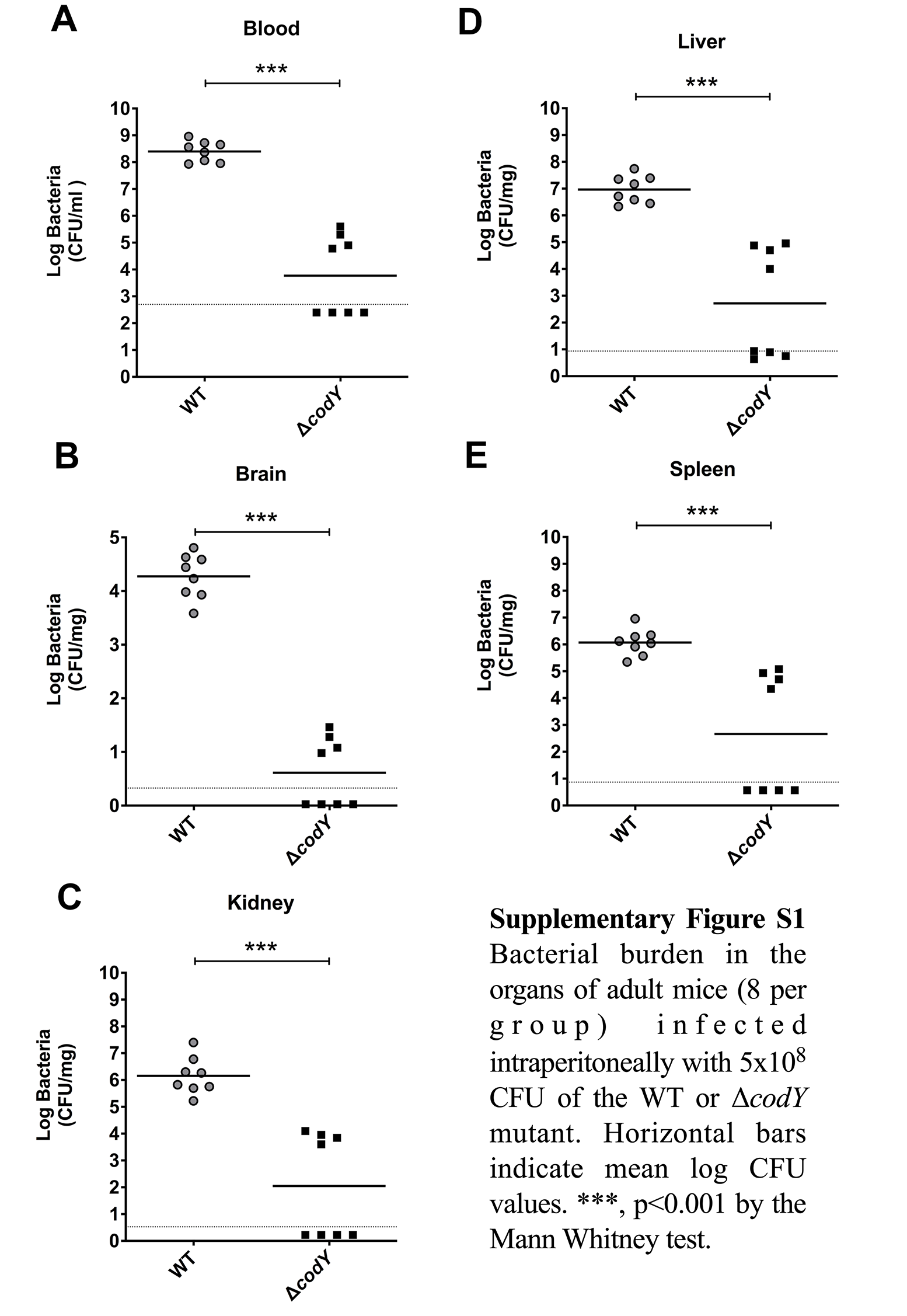

Supplement: Supplementary file 1 [file Image_1.tiff]

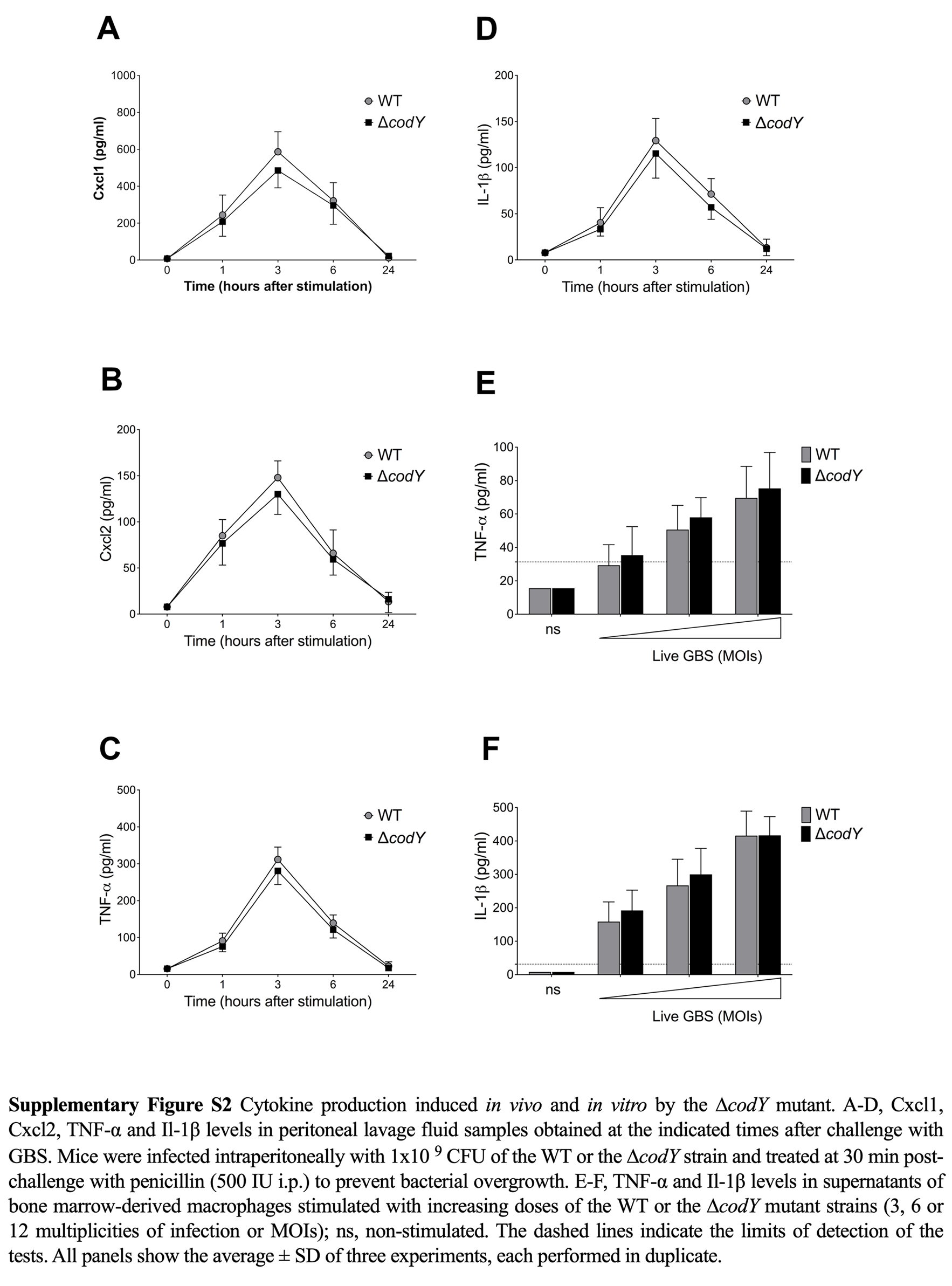

Supplement: Supplementary file 2 [file Image_2.TIFF]

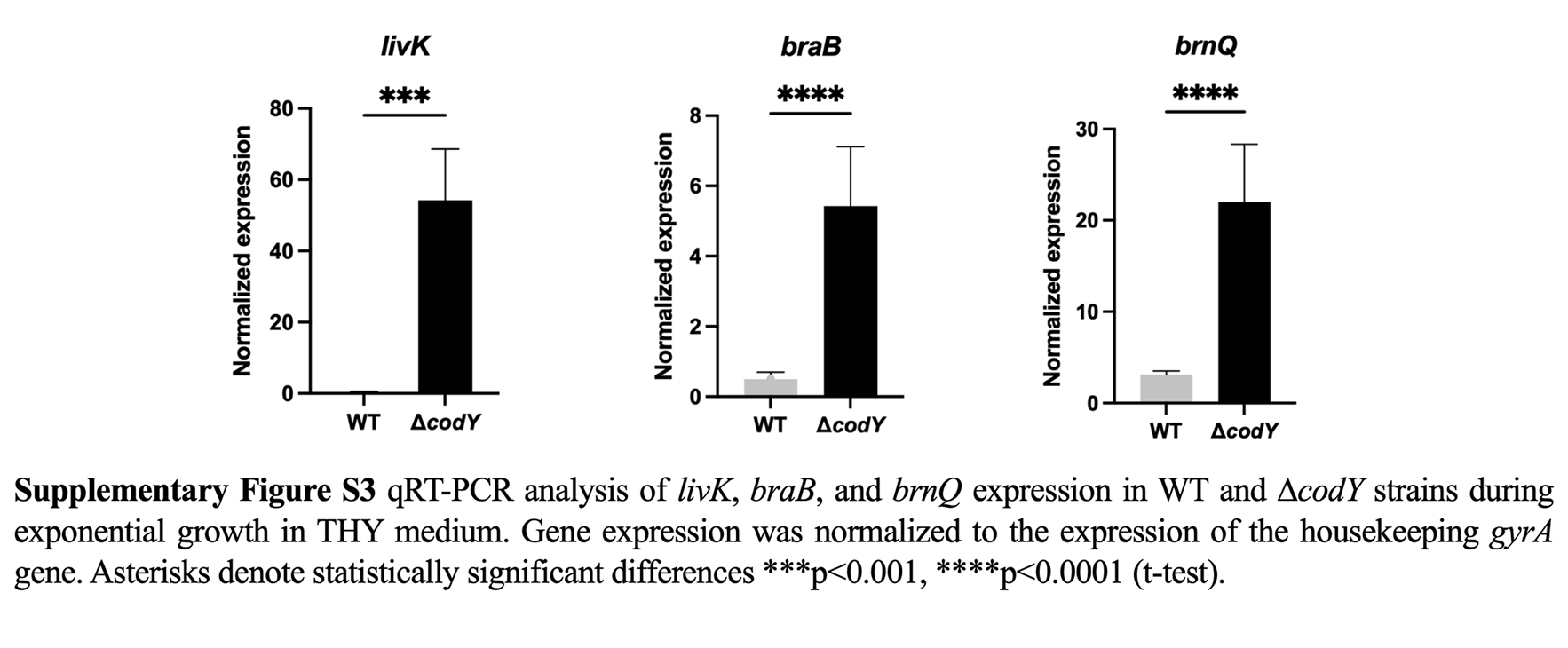

Supplement: Supplementary file 3 [file Image_3.TIFF]
