# Supplementary material for: CodY Is a Global Transcriptional Regulator Required for Virulence in Group B Streptococcus
Source: Front Microbiol. 2022 Apr 28;13:881549. doi: 10.3389/fmicb.2022.881549 (PMC9096947; doi:10.3389/fmicb.2022.881549)
Supplement: Supplementary file 6 [file Table_1.docx]

**Supplementary Table S1.** List of plasmids used in this work

| **Plasmid** | **Resistance** | **Relevant properties** | **Source or Reference** |
| --- | --- | --- | --- |
| pG1 | Erythromycin | This plasmid contains a temperature sensitive origin of replication for GBS and a ColE1 origin of replication for *E. coli*. | (Devaux et al., 2018) |
| pG1-Δ*codY* | Erythromycin | pG1 carrying the *codY* deletion cassette | This study |
| pTCVΩP_tet_ | Kanamycin/ Erythromycin | GBS expression vector carrying a constitutive P_tet_ promoter | (Firon et al., 2013) |
| pTCVΩP_tet__*codY* | Kanamycin/ Erythromycin | pTCVΩP_tet_ containing the full length *codY* ORF cloned between the BamHI/PstI restriction sites | This study |
| pTCV-*lacZ* | Kanamycin/ Erythromycin | Vector for the construction of transcriptional fusions to β-galactosidase in Gram-positive bacteria | (Poyart and Trieu-Cuot, 2006) |
| pTCV-*lacZ*_*livKp220* | Kanamycin/ Erythromycin | pTCV-*lacZ* carrying a 220 bp sequence which comprises the regulatory region of the *livK* gene cloned between the EcoRI/BamHI restriction sites of the plasmid. | This study |
| pTCV-*lacZ*_*livKp1-220* | Kanamycin/ Erythromycin | pTCV-*lacZ*_*livKp220* carrying a double nucleotide substitution mutation in one of the two CodY motifs located upstream of *livK*. | This study |
| pET28a | Kanamycin |  | Novagen |
| pET28a-*codY* | Kanamycin | pET28a carrying the full length *codY* ORF | This study |

Devaux, L., Sleiman, D., Mazzuoli, M.-V., Gominet, M., Lanotte, P., Trieu-Cuot, P., et al. (2018). Cyclic di-AMP regulation of osmotic homeostasis is essential in Group B *Streptococcus*. *PLoS Genet* 14, e1007342. doi:10.1371/journal.pgen.1007342.

Firon, A., Tazi, A., Da Cunha, V., Brinster, S., Sauvage, E., Dramsi, S., et al. (2013). The Abi-domain protein Abx1 interacts with the CovS histidine kinase to control virulence gene expression in group B *Streptococcus*. *PLoS Pathog* 9, e1003179. doi:10.1371/journal.ppat.1003179.

Poyart, C., and Trieu-Cuot, P. (2006). A broad-host-range mobilizable shuttle vector for the construction of transcriptional fusions to β-galactosidase in Gram-positive bacteria. *FEMS Microbiology Letters* 156, 193–198. doi:10.1111/j.1574-6968.1997.tb12726.x.
