# Supplementary material for: CodY Is a Global Transcriptional Regulator Required for Virulence in Group B Streptococcus
Source: Front Microbiol. 2022 Apr 28;13:881549. doi: 10.3389/fmicb.2022.881549 (PMC9096947; doi:10.3389/fmicb.2022.881549)
Supplement: Supplementary file 7 [file Table_2.docx]

**Supplementary Table S2.** List of primers used

| **Name** | **Sequence 5’-3’** |
| --- | --- |
| **Primers used in qRT-PCR experiments** | |
| braBF | TCTGGTGCTATCGCTACATTTC |
| braBR | CCCACCGTCTATTGGAGTATTG |
| brnQF | TGTGCCTAGTAGGAGGGTAATA |
| brnQR | TTCCACCAGCATTAGGTGTAG |
| livKF | CTCCGCTGGTGATACTGATTT |
| livKR | CGGTATAGTAACCTGGCATCAC |
| gyrAF | AAAGGGTCGTGGTGGTAAAG |
| gyrAR | TAATCGTCACTAAGCGTGCTAATG |
| **Primers used for the preparation of *lacZ* transcriptional fusions** | |
| livKp220F | CAAAATAGATATGAACAAATGAATTCGATATTGATCAGGATTTTGTTGGA |
| livKp220R | GTGTATCAACAAGCTGGGGATCCCGCTCCTAAACTAAGTCTCTTTTCCA |
| livKp1F | CAATTTAAAAACTATTGACAATATTCTCCTAATTCTGTATTATTTTAGTTAC |
| livKp1R | GTAACTAAAATAATACAGAATTAGGAGAATATTGTCAATAGTTTTTAAATTG |
| **Primers used for fragment labeling in EMSA experiments** | |
| Vlac1-FAM | GTTGAATAACACTTATTCCTATC |
| Vlac2-FAM | CTTCCACAGTAGTTCACCACC |
| **Primers used for BM110*codY* construction** | |
| COH1_1525FUp | GCTGGACACGGCTTTTATGATTACG |
| BM_codYFusR | CGCAAAACATACAAAGGATGAAGGAaTCATAGCATGGGACTGGGAGTAGC |
| BM_codYFusF | GCTACTCCCAGTCCCATGCTATGAtTCCTTCATCCTTTGTATGTTTTGCG |
| COH1_1527RDw | CCCAATTGCTTCTATTGCAGAGG |
| pG1R | GAATTCGTAATCATGGTCATAG |
| pG1F | GAGCTCGGTACCCGGGGA |
| pG1_codYUpF | ATGACCATGATTACGAATTCGTTAGCTAACATGAGGCTG |
| pG1_BM_codYDwR | GATCCCCGGGTACCGAGCTCCATTTAAGCTGGCTACAGC |
| **Primers used for *codY* complementation** | |
| pTCV_codYF_Bam | TGATGGATCCCGCAAAACATACAAAGGATGAAGGAA |
| pTCV_codYR_Pst | TGATCTGCAGGCTACTCCCAGTCCCATGCTATGA |
| **Primers used for *codY* cloning in pET28a** | |
| pET_GBS_codYF | AGCAAATGGGTCGCGGATCCATGCCGAATTTATTAGAAAAAAC |
| pET_GBS_codYR | TGTCGACGGAGCTCGAATTCTTAATTATATTCTTTTAATTTGTCAAAAATACC |
