# Supplementary material for: CodY Is a Global Transcriptional Regulator Required for Virulence in Group B Streptococcus
Source: Front Microbiol. 2022 Apr 28;13:881549. doi: 10.3389/fmicb.2022.881549 (PMC9096947; doi:10.3389/fmicb.2022.881549)
Supplement: Supplementary file 8 [file Table_3.DOCX]

**Supplementary Table S3.** Chemically defined medium composition

| **Component** | **Concentration** |
| --- | --- |
| Fe (NO_3_)_3_·9H_2_O | 2,5 mM |
| FeSO_4_·7H_2_O | 18 mM |
| MgSO_4_·7H_2_0 | 3 mM |
| MnSO_4_·H_2_O | 0.03 mM |
| NaC_2_H_3_O_2_·3H_2_0 | 33 mM |
| CaCl_2_ | 46 μM |
| L-cysteine HCl | 40 μM |
| NaHCO | 30 mM |
| glucose | 10 g/l |
| **Phosphate stock (10 X)** | |
| K_2_HPO_4_ | 11 mM |
| KH_2_PO_4_ | 74 mM |
| NaH_2_PO_4_·H_2_O | 0.6 M |
| Na_2_HPO_4_·7H_2_O | 1.3 M |
| **Bases stock (100 X)** | |
| Adenine | 2 mg/ml |
| Guanine hydrochloryde | 2 mg/ml |
| Uracil | 2 mg/ml |
| **Aminoacids stock (50 X)** | |
| DL-alanine | 5 g/L |
| L-arginine | 5 g/L |
| L-aspartic acid | 5 g/L |
| L-asparagine | 5 g/L |
| L-cystine | 2.5 g/L |
| L-glutamic acid | 5 g/L |
| L-glutamine | 10 g/L |
| Glycine | 5 g/L |
| L-histidine | 5 g/L |
| L-lysine | 5 g/L |
| L-methionine | 5 g/L |
| L-phenylalanine | 5 g/L |
| L-proline | 5 g/L |
| L-serine | 5 g/L |
| Hydroxy-L-proline | 5 g/L |
| L-serine | 5 g/L |
| L-threonine | 10 g/L |
| L-tryptophan | 5 g/L |
| L-tyrosine | 5 g/L |
| **Vitamins stock (1000 X)** |  |
| p-aminobenzoic acid | 0.2 mg/ml |
| Biotin | 0.2 mg/ml |
| Folic acid | 0.16 mg/ml |
| Niacinamide | 0.2 mg/ml |
| b-nicotinamide adenine dinucleotide phosphate | 0.5 mg/ml |
| Pantothenate calcium salt | 0.4 mg/ml |
| Pyridoxal | 0.2 mg/ml |
| Pyridoxamine dihydrochloride | 0.2 mg/ml |
| Riboflavin | 0.4 mg/ml |
| Thiamine hydrochloryde | 0.2 mg/ml |
| Vitamin B12 | - 1. g/ml |

L-isoleucine, L-leucine and L-valine were prepared separately as a 5 g/L stock and added to CDM medium at proper concentrations.

All components were purchased from Sigma (St. Louis, USA).
